# Marine Migrastatics: A Comprehensive 2022 Update

**DOI:** 10.3390/md20050273

**Published:** 2022-04-19

**Authors:** Marzia Vasarri, Emanuela Barletta, Donatella Degl’Innocenti

**Affiliations:** 1 Department of Experimental and Clinical Biomedical Sciences, University of Florence, Viale Morgagni 50, 50134 Florence, Italy; emanuela.barletta@unifi.it (E.B.); donatella.deglinnocenti@unifi.it (D.D.); 2 Interuniversity Center of Marine Biology and Applied Ecology “G. Bacci” (CIBM), Viale N. Sauro 4, 57128 Livorno, Italy

**Keywords:** marine compounds, migrastatics, cell migration, cell invasion, metastasis, anti-cancer

## Abstract

Metastasis is responsible for the bad prognosis in cancer patients. Advances in research on metastasis prevention focus attention on the molecular mechanisms underlying cancer cell motility and invasion to improve therapies for long-term survival in cancer patients. The so-called “migrastatics” could help block cancer cell invasion and lead to the rapid development of antimetastatic therapies, improving conventional cancer therapies. In the relentless search for migrastatics, the marine environment represents an important source of natural compounds due to its enormous biodiversity. Thus, this review is a selection of scientific research that has pointed out in a broad spectrum of in vitro and in vivo models the anti-cancer power of marine-derived products against cancer cell migration and invasion over the past five years. Overall, this review might provide a useful up-to-date guide about marine-derived compounds with potential interest for pharmaceutical and scientific research on antimetastatic drug endpoints.

## 1. Introduction

Worldwide, cancer ranks among the leading causes of death. An estimated 19.3 million new cases occurred in 2020, with a mortality rate exceeding 50% [1]. Cancer develops due to complex biological processes such as uncontrolled cell proliferation, resistance to cell death, neo-angiogenesis, invasion, and metastasis [2].

The most common cause of cancer-related death is invasion and metastasis, in which cancer cells spread throughout the body, causing secondary sites of invasion and severe organ damage [3].

Significant progress has been made in treating malignant tumors thanks to the development of new technologies and combination therapies used as multiple approaches to affect several signaling pathways simultaneously [4]. However, traditional cytotoxic chemotherapeutic agents are still the most commonly used. Conventionally, the progression of solid cancers is defined by the increase in tumor size. Therefore, it is understandable that tumor shrinkage is considered one of the indices of therapeutic efficacy. However, tumor shrinkage is never absolute and is not predictive of antimetastatic effect and may divert attention from cancer metastasis, which is responsible for more than 90% of mortality [5].

Therefore, blocking cancer cell migration and the possibility of secondary tumor formation is crucial to significantly improve cancer treatment by eliminating the risk of systemic disease and reducing reliance on therapies with harmful side effects. Combining antimetastatic therapy with standard cytotoxic cancer therapies appears to be a promising approach for treating metastases [6,7].

In light of these considerations, Gandalovičová et al. (2017) coined the term “migrastatics” to define a new class of drugs capable of interfering with cancer cell migration and invasion, and thus with the ability to metastasize [5]. Migrastatic agents, which differ from conventional cytostatic compounds that primarily target cell proliferation, are important for treating cancer with a propensity for early metastasis. This new definition shifts the focus from primary cancer therapy to the treatment of metastasis development and from tumor shrinkage as a measure of treatment efficacy to metastasis-free survival as the most representative endpoint for antimetastatic drugs [5].

Despite the large arsenal of anti-cancer drugs available, inadequate selectivity toward cancer cells and developed resistance account for the serious side effects of therapy. This has made it more urgent than ever to find new drugs, and for this purpose, it has often proven useful to consider the pharmacological properties of natural compounds. To date, the majority (at least 60%) of anti-cancer drugs are derived from natural products of both terrestrial and marine origin. However, unlike the terrestrial environment, marine biodiversity represents a vast and largely unexplored source of bioactive compounds characterized by structural novelty, complexity, and diversity. The unique conditions of life in the marine environment require special mechanisms for organisms to adapt to growth in the ocean that are fundamentally different from those of terrestrial organisms. One important adaptation mechanism is the production of biologically active secondary metabolites. Secondary metabolites are not produced using biological or regular metabolic pathways and do not serve any primary function relevant to the development, growth, or propagation of a species. However, they provide a wealth of chemical diversity potentially useful for drug development because of their biological activities [8].

In addition, continued improvements in undersea life support systems have provided new opportunities for harvesting from unexplored regions and depths. The sea’s treasures have made fundamental contributions to modern medicine, providing important scientific discoveries for human health [9]. Thus, marine bio-discovery has become a frontier pursuit for many scientists in academia and industry in recent decades. Continued exploration of the marine environment and characterization and screening of its inhabitants for unique bioactive chemicals has already proven fruitful in the search for new anti-cancer therapies [10,11]. Clinical trials are underway for a large number of marine-derived anti-cancer compounds [12], and the field is currently expanding rapidly.

It is conceivable that many marine species have yet to be discovered, which could lead to the identification of a myriad of marine natural products that could enhance and complement existing anti-cancer therapies. The literature is full of reviews on natural anti-cancer products and their various mechanisms of action, but what are the latest findings on marine anti-cancer agents with migrastatic potential? Here is a selection of marine anti-cancer compounds described in the last five years (coining of the term “migrastatics”) for their ability to inhibit cancer cell migration and invasion. This review represents a useful tool for pharmaceutical industries and basic biomedical science to improve the knowledge of marine migrastatics as potential candidates for developing new anti-cancer strategies.

## 2. Biomechanisms Underlying Cancer Metastasis as Therapeutic Molecular Targets

**Figure 1:**
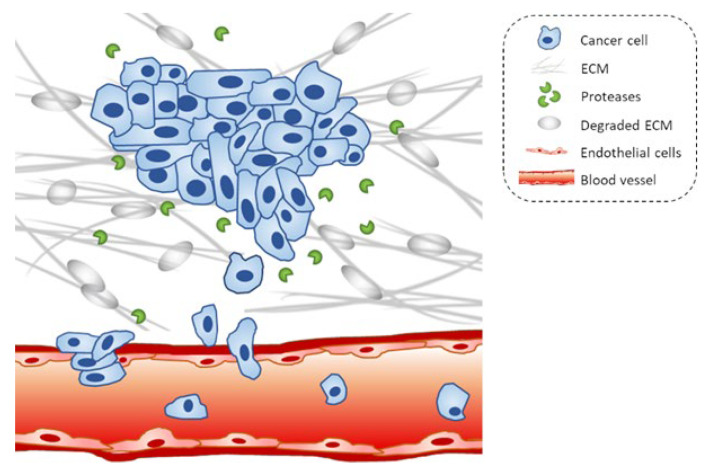
Schematic representation of the migration and invasion process of cancer cells. Cells penetrate the basement membrane and invade surrounding tissues; cancer cells invade blood vessels and give rise to metastasis.

Metastasis originates from the spread of tumor cells into tissues and organs beyond their primary site and their establishment at secondary sites with the formation of new tumors [13]. The first step in the metastatic process is the migration of cancer cells through the extracellular matrix (ECM) of the surrounding tissue and penetration into the lymphatic or vascular circulation. After the extravasation process through the basement membrane of the vessels, the invasive cells will attach to a new site and proliferate to produce the secondary tumor [3] (Figure 1).

The dynamics of this process depend on multiple biomechanisms and signaling pathways that promote the metastatic progression of cancer cells. Understanding these biomechanisms is essential to identifying molecular targets to reverse the pro-metastatic behavior of cancer cells.

Below we will briefly present the biomechanisms underlying metastasis described as potential druggable targets of marine migrastatics over the past five years (2017–2022).

### 2.1. Epithelial-Mesenchymal Transition (EMT) and EMT-Related Signaling Pathways

The invasiveness and metastatic potential of malignant epithelial tumors, which represent about 90% of all cancer cases, are supported by the so-called epithelial-mesenchymal transition (EMT). This is a process by which epithelial cells acquire fibroblastoid properties [14]. During EMT, there is loss of cell-cell adhesion, cytoskeletal remodeling, and alteration in cell-ECM adhesion. This process is triggered by complex regulatory networks involving transcriptional control within the affected epithelial cells, such as the suppression of E-cadherin (a known epithelial marker) expression by key regulators of EMT (such as Snail, Slug, and Twist) [15,16]. As a result, cells become more motile and adhesive to endothelial cells. Furthermore, the EMT process is regulated by many molecular events, including multiple signaling pathways in various cancers [14]. Among them, the Hedgehog (Hh) signaling pathway has been described to play a role in regulating EMT [17,18,19,20]. Aberrant activation of the Hh signaling pathway has been associated with cancer onset and metastasis formation [21]. Thus, EMT and related signaling pathways are meaningful targets for blocking the pro-metastatic behavior of cancer cells.

### 2.2. ECM Disassembly and the Role of Matrix Metalloproteinases (MMPs)

Cancer cell migration and invasion are also influenced by ECM disassembly. The ECM is a source of cell-binding proteins and growth factors important to tumor cell survival. ECM can be modified significantly by proteases generated by tumor cells or stromal cells. These ECM proteases affect the cell-cell and cell-ECM interactions. Among ECM proteases, matrix metalloproteinases (MMPs)—a family of zinc-dependent endoproteinases—play an important role as early markers in cancer progression. They facilitate invasion by degrading components of the ECM, such as collagen, laminin, and proteoglycans [22]. However, it has become clear that MMPs play a role in ECM modification and regulate numerous other biological events, including cell growth, inflammation, and angiogenesis, by cleaving cell surface proteins and adhesion molecules [23]. Among all members of the MMPs, it has been reported that MMP-2 and MMP-9 correlate with tumor metastasis [24]. Therefore, targeting the expression and/or activity of MMPs or other ECM proteases has long been considered promising targets for cancer therapy.

### 2.3. Anoikis Resistance

As part of normal development and tissue homeostasis, a cell death called anoikis (detachment-induced apoptosis) acts to prevent detached cells from reattaching to the wrong ECM and growing dysplastically. During the detachment of ECM-anchored cells, anoikis is triggered [25]. A tumor cell exhibiting pro-metastatic behavior develops altered cell adhesion mechanisms and anoikis resistance. A detached cell from a localized tumor can escape anoikis, resulting in uncontrolled growth. Cancer cells employ many mechanisms to abrogate anoikis, promoting invasion and metastasis [26]. By providing adequate structural support, ECM-based adhesion is crucial for cell survival. Several factors have been identified for modulating anoikis resistance, such as cell adhesion molecules, growth proteins, oxidative stress, stemness, autophagy, non-coding RNAs, and signaling pathways [26].

### 2.4. Cytoskeletal Remodeling and Cell Adhesion

The formation of membrane protrusions is essential for cancer cell migration in response to migratory and chemotactic stimuli. The polymerization of submembrane actin filaments is the driving force behind membrane protrusions that provide rigidity to cancer cells. Filiform structures, generated by the cytoskeletal remodeling of submembrane actin filaments, are critical factors for metastatic cancer cells to adhere to secondary tissues/organs. Through the filopodia, one of the filiform structures, metastatic tumor cells play an important role in signal sensing and cancer cell adhesion, as well as migration and invasion [27,28]. Promoting cell detachment from the ECM by limiting filopodia formation and thus inhibiting cell adhesion could improve cell anoikis and be a valuable target in anti-metastatic therapy.

Focal adhesions, i.e., clustering sites of integrins, are vital for cell survival. Connecting the actin cytoskeleton to the ECM provides a physical attachment of cells to the ECM. Thus, integrins, important cell adhesion receptors, are implicated in almost all stages of cancer progression from primary tumor development to metastasis. Numerous pieces of evidence draw attention to the function of focal adhesion kinase (FAK)—a non-receptor tyrosine kinase—in mediating inside-out signaling of focal adhesion to regulate cell proliferation, survival, and migration [29]. Therefore, targeting integrin and its downstream signaling could help antimetastatic treatment.

The coordination of dynamic reorganization between actin filaments and microtubules is critical for the transmigration of cancer cells through the vasculature and their metastasis. Microtubules exist as a component of the cytoskeleton, and they are important for maintaining the physical and plastic properties of migrating cells and random movement. Therefore, microtubule-interfering agents could serve as potential antimetastatic agents [30].

### 2.5. Hypoxia and Neo-Angiogenesis

Oxygen plays an important role in regulating developmental processes and tissue homeostasis. At the cellular level, oxygen is required for oxidative metabolism and cell survival. Therefore, hypoxia and hypoxic signaling are potent contributing factors to tumor progression to metastasis [31].

The rapid proliferation of cancer cells results in a high oxygen consumption in the tumor microenvironment. Oxygen deprivation in the tumor causes apoptosis and necrosis. Briefly, hypoxia triggers the response of hypoxia-inducible factors (HIFs), transcriptional factors that coordinate an adaptive cellular response to low oxygen levels. Additionally, hypoxia triggers the release of angiogenic growth factors to restore a functional vascular system. In particular, HIF-1α promotes the late phases of metastasis in the distant site by stimulating neo-angiogenesis through the up-regulation of vascular endothelial growth factor (VEGF) [31,32]. Thus, neo-angiogenesis-targeted therapy might strike cancer cells in addition to its effect on vasculature.

### 2.6. Apoptosis and Autophagy

Apoptosis and autophagy are physiological cell death processes that can counteract tumor progression. Apoptosis is programmed cell death that leads to the removal of damaged cells. Apoptosis can block metastatic dissemination by killing misplaced cells resulting in an important process for limiting metastasis formation [33,34]. Deregulation of apoptosis is recognized as a hallmark of cancer as it promotes tumor progression and resistance to therapy [35].

Autophagy is a process that involves the action of lysosomes to degrade damaged organelles and macromolecular substances. Although the role of autophagy in cancer remains controversial, autophagy has been reported to act in an antimetastatic role by limiting cancer necrosis and inflammatory responses in the early stages of cancer metastasis. In early metastasis, autophagy also reduces the migration and invasion of cancer cells from the sites of origin [36]. Therefore, the regulation of apoptosis and autophagy is a potential approach to cancer advancement.

### 2.7. Epigenetic Control

Metastatic traits are increasingly thought to derive from the epigenetically modified transcriptional output of oncogenic signals that initiate and drive tumor development. Evidence points to a broad role for epigenetic mechanisms in key steps of metastasis, including DNA methylation, histone modifications, nucleosome changes, and non-coding RNAs [37]. Unlike genetic abnormalities, epigenetic changes are reversible and thus potential druggable targets for anti-cancer strategies. Epigenetic therapies thus have great potential to reprogram cancer cells and thereby halt the advancement of cancer [38].

## 3. Marine Compounds as Migrastatics

Marine bioactive compounds have attracted a great deal of interest in human health. Human health research has long been concerned with exploring and identifying natural products that can be useful in designing and developing novel therapeutic approaches against cancer [39].

In light of growing evidence that cell migration is crucial to cancer development, scientific research seeks new molecules that could prevent cancer cells from spreading.

**Figure 2:**
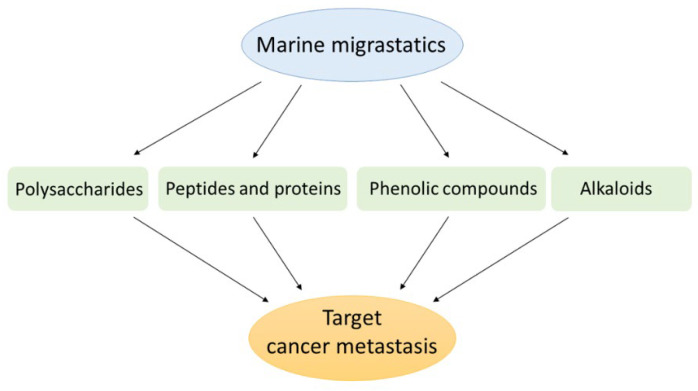
Bioactive compounds from marine entities as migrastatics for anti-cancer application.

For years, researchers and experts have navigated the molecular treasures of the sea to search for and identify structurally novel and biologically active metabolites of natural products. The following sections review the last five years of research on new or known marine compounds for which a migrastatic role has been described and then proposed to the scientific community as potential agents for cancer therapy. As reported in Figure 2, the discussed bioactive marine compounds belong to several categories, namely polysaccharides, peptides and proteins, phenolic compounds, and alkaloids.

### 3.1. Marine Polysaccharides

Sulfated polysaccharides such as carrageenan, fucoidan, and laminaran from various marine organisms and microorganisms have shown potent anti-migratory activity against several in vitro and vivo cancer models.

Exopolysaccharide 11 [EPS11] from the marine bacterium *Bacillus* sp. 11. was found to inhibit the migration of human lung cancer A549 cells by destroying filiform structures [40]. These structures are organelles widely found in cancer cells, which promote the invasion and migration of cancer cells. By the same mechanism, EPS11 was found to inhibit the migration of human hepatoma Huh7.5 [41], HepG2 and Bel-7402 cells [42] by acting on the cell membrane surface, i.e., destroying filiform structures and down-regulating the expression of CD99, an essential factor for cell adhesion, migration and cancer metastasis. Moreover, it has been revealed that EPS11 inhibits the metastatic process in vivo in animal models of metastatic melanoma [41].

Algae are an important source of polysaccharides with diverse biological activities among marine organisms. In this context, Ou et al. (2017) demonstrated the migrastatic potential of the water-soluble fucoidan-like polysaccharide STPC2 isolated from the brown alga *Sargassum thunbergii*. This polysaccharide was able to significantly inhibit the migration of lung cancer cells A549 via downregulation of MMP-2 activity—which is associated with cancer metastasis—and affect cell viability at a high concentration [43].

Laminaran sulfate isolated from the brown alga *Fucus evanescens* was shown to be a potent anti-migratory agent by inhibiting MMP-2 and MMP-9 activity in human colorectal adenocarcinoma (HCT116 cells), malignant melanoma (SK-MEL-5 cells), and breast adenocarcinoma (MDA-MB-231 cells) [44].

Malyarenko and colleagues (2019) also conducted a study that demonstrated for the first time the ability of sulfated laminaran from the brown alga *Dictyota dichotoma* to significantly increase the inhibitory effect of X-rays on SK-MEL-28 melanoma cell migration by regulating MMP-2 and MMP-9 activity. These results may provide a new combination strategy for radiation treatment in oncology [45].

Migrastatic properties have also been described for sulfated polysaccharides (SPUP) from the brown alga *Undaria Pinnatifida*. In particular, it could inhibit the migration of MCF7 breast cancer cells [46] and SKOV-3 and A2780 ovarian cancer cells by suppressing the Hedgehog signaling pathway [47].

Preventive effects against the migratory and invasive behavior of A2780 ovarian cancer cells were also attributed to a novel water-soluble polysaccharide (BFP-3) [48] isolated from the red alga *Bangia fuscopurpurea* by Wu et al. (2021). The migrastatic action of BFP-3 was likely due to mechanisms that promote apoptotic and autophagic cell death through the mitochondria-dependent pathway [49].

Fucoidan from the sea cucumber *Cucumaria frondosa* has been reported to inhibit in vitro migration of human osteosarcoma U2OS cells by impairing dynamic cytoskeletal remodeling through the suppression of the phosphorylation of FAK and paxillin and activation of the Rac1/PAK1/LIMK1/cofilin signaling axis [50].

The sulfated polysaccharide (SIP-SII) from *Sepiella maindroni* ink was shown to inhibit EGF-induced migration and invasion of human epidermoid carcinoma KB cells [51] and human ovarian carcinoma SKOV-3 cells [52] by suppressing the epidermal growth factor receptor (EGFR)-mediated Akt and p38 MAPK cascades and subsequently inhibiting MMP-2 expression.

Reduced phosphorylation of EGFR is linked to phosphorylation of ERK1/2, which is the downstream effector of FAK and E-cadherin. Up-regulation of FAK and down-regulation of E-cadherin are crucial in promoting cancer cell migration. In this context, sulfated galactan (SG) [53], from the red alga *Gracilaria fisheri*, suppresses HuCCA-1 cholangiocarcinoma cell migration by downregulating the EGFR-ERK signaling pathway. Specifically, SG inhibits ligand-induced EGFR dimerization by binding itself to the dimerization arms of unbound EGFR [54]. The up-regulation of FAK and down-regulation of E-cadherin are crucial to promoting cancer cell migration. Reduced phosphorylation of EGFR is related to the phosphorylation of ERK1/2, which is the downstream effector of FAK and E-cadherin [55].

Fractions rich in sulfated polysaccharides have also been attributed to migrastatic activity, such as those extracted from the green alga *Caulerpa cupressoides* var. *flabellata*, which inhibit the migration of B16-F10 melanoma cells [56]. Crude sulfated polysaccharides isolated and purified from the brown algae *Padina tetrastromatica* have been shown to block the migration of human cervical adenocarcinoma HeLa cells. They inhibit cell migration by lowering the expression of MMP-2 and MMP-9 and hinder angiogenesis through the downregulation of angiogenic mediators VEGF and HIF-1-α expression [57].

Table 1 summarizes all the above-mentioned marine polysaccharides as migrastatics.

### 3.2. Marine Peptides and Proteins

In recent decades, natural marine peptides have attracted much attention because of their specific characteristics, including a broad spectrum of bioactivity and low toxicity, making them very promising candidates against a wide spectrum of diseases, including cancer. Today, several anti-cancer peptides and their derivatives from marine organisms and microorganisms have been widely applied to clinical research [11,58,59,60].

Peptides are molecules that participate in all processes of life activities. They can have anti-tumor roles in diverse aspects in different ways, such as preventing cell migration.

Several depsipeptides (nonribosomal peptides cyclized via an ester bond and often containing nonprotein amino acids) have been approved to treat various cancers or are undergoing clinical trials for potential utility as anti-cancer drugs [61].

**Figure 3:**
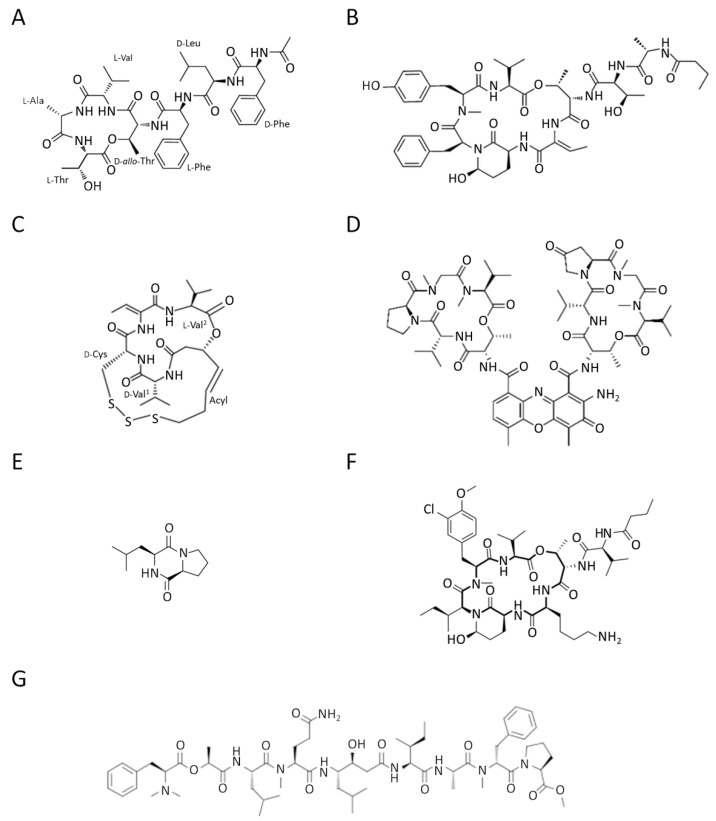
Chemical structure of (**A**) nobilamide I from the bacterium *Saccharomonospora* sp., strain CNQ-490; (**B**) molassamide from the Cyanobacterium DRTO-73; (**C**) chromopeptide A from the bacterium *Chromobacterium* sp. HS-13-94; (**D**) actinomycin V from *Streptomyces* sp.; (**E**) cyclo(L-leucyl-L-prolyl from marine microorganisms; (**F**) kempopeptin C from the cyanobacterium *Lyngbya* sp., (**G**) grassystatin F from the Cyanobacterium VPG 14-61.

A recent study isolated a novel cyclic depsipeptide, nobilamide I, from the marine-derived bacterium *Saccharomonospora* sp., strain CNQ-490 (Figure 3A). This showed significant migrastatic properties in three different cancer cell lines, lung cancer A549 cells, gastric cancer AGS cells, and colorectal cancer Caco2 cells, by modulating the expression levels of MMP-2/9 [62].

Molassamide (Figure 3B), isolated from the marine cyanobacterium DRTO-73, showed a dual action on the migration of highly invasive MDA-MB-231 breast cancer cells by modulating both elastase-induced intercellular adhesion molecule (ICAM)-1 gene expression and ICAM-1 proteolytic processing by elastase [63].

Chromopeptide A (Figure 3C) is a depsipeptide isolated from the marine-derived bacterium *Chromobacterium* sp. HS-13-94 [64]. In in vitro and in vivo models of prostate cancer (PC3 cells), chromopeptide A showed effective anti-migratory and anti-metastatic properties by acting as an inhibitor of class I histone deacetylases (HDACs). It is well known that histone deacetylases are involved in posttranslational modification of gene expression [65]. Therefore, chromopeptide A, by affecting HDACs, can play a role in epigenetic mechanisms of regulation of several biological events. Notably, the peptide showed in vivo better body weight tolerance than FK228 (already FDA approved), implying controllable toxicity. These data capture attention for the potential applications of the marine-derived peptide in prostate cancer therapy [66]. HDACs play an important role in EMT regulation in a variety of cancers. It has been reported that treatment of cells with an HDAC inhibitor represses EMT and metastasis [67].

Actinomycin V (Figure 3D) is a cyclic chromopeptide produced by the marine actinomycete *Streptomyces* sp. Lin and colleagues reported a suppressive effect of actinomycin V on the migration and invasion of human breast cancer MDA-MB-231 cells. This effect may be explained by inhibiting EMT through the down-regulation of Snail and Slug protein expression [68].

The cyclic dipeptide cyclo(L-leucyl- L-prolyl) (CLP) from various marine microorganisms has a myriad of pharmaceutical significances (Figure 3E). Deepak Kgk and colleagues have demonstrated that CLP suppresses growth and migration by attenuating the cell cycle of MDA-MB-231 and MDA-MB-468 triple-negative breast cancer cells via CD151/EGFR signaling [69].

Kempopeptin C depsipeptide (Figure 3F), a protease inhibitor from the marine cyanobacterium *Lyngbya* sp., was also shown to suppress the migration of invasive MDA-MB-231 breast cancer cells [70].

Grassystatin F (Figure 3G), isolated from the marine cyanobacterium VPG 14-61, also acts as a protease inhibitor. This potent aspartic protease inhibitor can inhibit the migration of highly aggressive MDA-MD-231 triple-negative breast cancer cells [71].

Table 2 summarizes the specific information on the migrastatic marine depsipeptides described above.

The marine abalone gastropod is a marine organism rich in vital elements, including proteins and peptides, and is known for its many biological properties [72]. Recent literature also describes a migrastatic role of some bioactive peptides obtained from boiled abalone (*Haliotis discus hannai*). In particular, BABP and AATP peptides inhibited the migration and invasion of human fibrosarcoma cells (HT1080) and human umbilical vein endothelial cells (HUVEC) by attenuating MMPs and VEGF [73,74].

An analysis of the activity of the αO-conotoxin GeXIVA from *Conus generalis* [75] in MDA-MB-157 breast cancer cells performed by Sun and colleagues revealed the inhibitory activity on cell migration of this conotoxin, probably downregulating α9-nAChR (α9 nicotinic acetylcholine receptor), essential for mediating tumor metastasis through EMT [76].

Enzyme proteins have also been described to have effects on cancer cell migration. This is the case of the N-V protease or Nereis active protease (NAP), which is a fibrinolytic active serine protease purified from the polychaeta *Nereis virens* [77]. An in vitro study conducted in 2019 demonstrated that NAP inhibited the migration of lung cancer H1299 cells probably by inducing apoptosis through the inhibition of the PI3K/AKT/mTOR pathway [78].

Adding to the list is the polypeptide PBN11-8, purified from the fermentation broth of *Bacillus* sp. N11-8. This, similar to the M84 peptidase from *Bacillus pumilus*, belonged to a type of zinc-dependent metalloprotease family. PBN11-8 was shown to suppress the invasion and migration of human hepatocellular carcinoma BEL-7402 cells by targeting the FAK pathways and MMP-2/9 [79].

Table 3 provides specific information about the peptides mentioned above and proteins with migrastatic action.

Not only single peptides or proteins but also crude protein extracts have demonstrated potent migrastatic bioactivity. This is the case of proteins extracted from sea cucumber (*Holothuria leucospilota*) that have shown a potential anti-cancer activity towards human hepatoma HepG2 cells, human lung carcinoma A549 cells, and human pancreatic cancer Panc02 cells, with a higher efficacy than a clinical anti-cancer drug [80].

### 3.3. Marine Phenolic Compounds

Polyphenols possess a variety of biological functions, making them one of the most important phytochemicals [81]. Several studies have shown natural polyphenols’ protective effects against chronic diseases and an inverse correlation between polyphenol consumption and cancer development [82]. Various polyphenols have also been shown to inhibit cancer growth via pleiotropic mechanisms targeting multiple signaling pathways in charge of key cellular processes, including cancer cell migration [82].

Among phenolic compounds, phloroglucinol and its derivatives, which exist in brown algae, have attracted attention because of their good biological activity [83]. In particular, the phloroglucinol derivative 7-phloroeckol (7PE), derived from the brown alga *Ecklonia cava*, has recently shown migrastatic abilities in hepatoma cancer HepG2 cells and anti-angiogenic properties on human umbilical vein endothelial HUVEC cells. Furthermore, 7PE has been shown to act through PI3K/AKT/mTOR and Ras/ERK signaling pathways to inhibit HIF-1α protein expression from blocking VEGF protein production [84].

In addition, the phlorotannins dieckol and phlorofucofuroeckol obtained from *Ecklonia cava* were shown to be able to block LPS-induced migration and invasion of human breast cancer cells (MCF-7 and MDA-MB-231 cells) by reducing both MMP-2/9 or TLR-4 expression and NF-κB transcriptional activity, which was induced by LPS [85].

Many benzaldehyde derivatives produced as secondary metabolites by marine-derived fungi have also been described for their bioactive properties [86]. In this context, the marine fungus *Eurotium chevalieri*—an endophyte of the sponge *Grantia compressa*—was the source of the prenylated benzaldehyde derivative dihydroauroglaucin (DAG). DAG was found to inhibit the migration of human SH-SY5Y neuroblastoma cells by activating autophagy [87].

Cell migration is dependent on microtubule dynamics [30], and viriditoxin from the marine-derived fungus *Paecilomyces variotii* [88] was found to reduce the ability of human ovarian SKOV-3 cells to migrate by disrupting microtube dynamics [89].

Polyphenol-rich fractions have also proved to be valid biological tools against the migration of cancer cells, probably exploiting the synergistic action of several constituents [90]. This is the case of the polyphenolic fraction obtained from the brown seaweed *Padina boergeseni*, of which gallic acid, caffeic acid, rutin, quercetin ferulic acid were the main components; this was able to significantly inhibit the migration of renal cancer cells (A498 and ACHN cells) by involving cell cycle arrest and inducing apoptosis [91].

Additionally, phenolic phytoconstituents extracted from the red algae *Gelidiella acerosa* showed migrastatic properties in lung adenocarcinoma A549 cells by decreasing the expression level of MMP-2 [92].

In addition, the hydroalcoholic extract obtained from the marine plant *Posidonia oceanica* was found to be a rich reservoir of phenolic compounds, including catechin, epicatechin, gallic acid, ferulic acid, and chlorogenic acid. This has been shown to inhibit the migration of human fibrosarcoma HT1080 cells [93] and human neuroblastoma SH-SY5Y cells [94] by acting on MMPs and activating autophagy. Furthermore, the polyphenol-rich phytocomplex is suitable for use as a vehicle in nanoformulations that can improve bioavailability and its migrastatic bioactivity [95].

All polyphenols or polyphenol-rich fractions presented here (Table 4) share potential applicability in anti-cancer therapies.

### 3.4. Marine Alkaloids

Marine organisms have been developed as a new source of naturally occurring alkaloids. Various alkaloids of marine origin have been reported to be potentially active against cancer metastasis [96].

**Figure 4:**
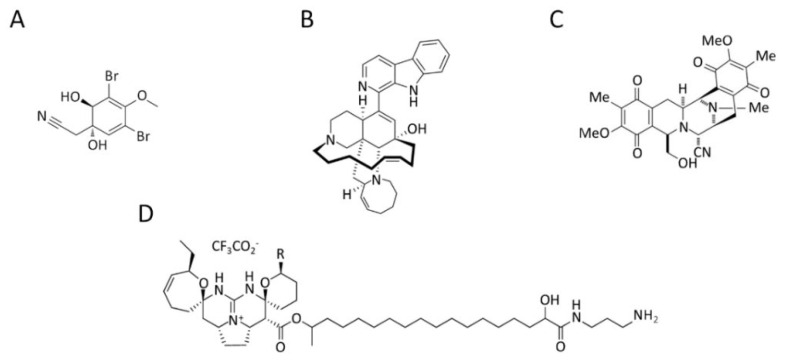
Chemical structure of (**A**) aeroplysinin-1 from the marine sponge *A. aerophoba*; (**B**) manzamine A from the sponge of the genera *Haliclona* sp., *Xestospongia* sp. and *Pellina* sp.; (**C**) jorunnamycin A from the sponge *Xestospongia* sp.; (**D**) normonanchocidines G (R=CH_3_) and H (R=H) from the marine sponge *M. pulchra*.

A brominated alkaloid, aeroplysinin-1 (Figure 4A), isolated from the marine sponge *Aplysina aerophoba,* showed decreased migration and adhesion of mouse pheochromocytoma MTT cells in vitro by downregulating integrin β1 [97].

In addition, manzamine A (Figure 4B), a pentacyclic β-carboline-fused alkaloid derived from a sponge of the genera *Haliclona* sp., *Xestospongia* sp. and *Pellina* sp., inactivates the EMT and the migratory capacity of human colorectal carcinoma HCT116 cells. This alkaloid suppresses several mesenchymal transcription factors (e.g., Snail, Slug and Twist) and simultaneously induces the epithelial marker E-cadherin leading to epithelial phenotype and migration suppression. This study showed that manzamine A is a potential migratory anti-cancer drug for colorectal cancer patients with tumors that undergo the EMT process and develop distal metastases [98].

In addition, the alkaloid jorunnamycin A (Figure 4C), a bistetrahydroisoquinolinequinone isolated from a Thai blue sponge *Xestospongia* sp. [99], showed the ability to inhibit EMT, sensitize anoikis, and suppress anchorage-independent survival in H460 human lung cancer cells. Since the loss of anchorage dependence is an important block to metastasis, jorunnamycin A could be an effective agent against lung cancer metastasis [100].

Two pentacyclic guanidine alkaloids, normonanchocidines G and H (Figure 4D), isolated from the marine sponge *Monanchora pulchra*, manifested a high impact on the capacity of human cervical epithelioid carcinoma HeLa [101].

Table 5 summarizes the specific information on the migrastatic marine alkaloids described above.

## 4. Research Methodology

This review selected literature on marine migrastatics described since 2017, when the “migrastatics” term was coined.

Extensive searches were performed in PubMed, ScienceDirect, Web of Science, and Google Scholar databases using one or more of the following terms: “marine anti-cancer compounds”, “marine anti-migratory compounds”, “marine anti-invasive compounds”, “marine compounds against metastasis”, “marine migrastatics”, and “migrastatics in cancer”.

A total of 42 articles describing marine migrastatics in 2017–2022 were selected and included. Pre-2017 literature on marine anti-migratory and anti-invasive compounds were excluded. Chemical structures were adapted from PubChem, and figures were created using Microsoft PowerPoint per Microsoft 365 MSO (Version 2202 Build 16.0.14931.20128).

## 5. Summary and Future Perspectives

Although nearly two decades have passed since the six hallmarks of cancer were defined, anti-cancer therapeutic strategies have not focused enough on the mechanism responsible for the highest mortality in patients with solid tumors: metastasis.

Cancer cells undergo complex modifications in cancer progression and achieve an invasive phenotype to become metastatic.

Research is continually working to find new anti-cancer drugs to limit the pro-metastatic behavior of cancer cells. Hopes and expectations for more specific and less toxic therapies are ever-present.

However, not all available anti-cancer drugs can curb metastatic tumor aggressiveness. In 2017, the term “migrastatic” was coined to distinguish a category of anticancer agents capable of preventing cancer cell migration and invasion.

In the relentless search for migrastatics, the marine environment represents an untapped source of natural compounds due to its enormous biodiversity.

**Figure 5:**
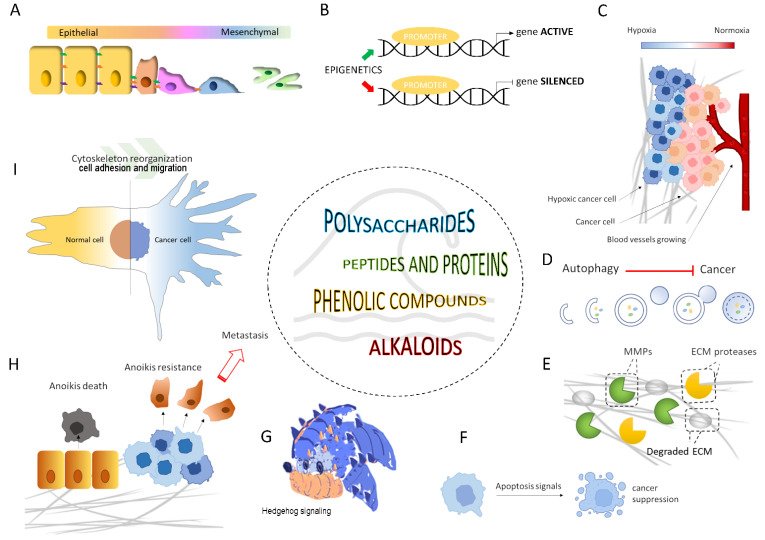
Schematic representation of potential molecular targets of marine migrastatics (2017–2022): (**A**) epithelial-mesenchymal transition; (**B**) epigenetic control; (**C**) neo-angiogenesis and hypoxia; (**D**) autophagy; (**E**) ECM degradation by MMPs and other proteases; (**F**) apoptosis; (**G**) Hedgehog signaling pathway; (**H**) resistance to anoikis; (**I**) cytoskeletal reorganization, cell adhesion and migration. In addition, as shown in Figure S1 of the Supplementary Materials, each of these pictorial representations of the molecular target for metastasis was linked to the migrastatic marine compounds described.

Data published in the past five years illustrate the migrastatic properties of compounds (e.g., polysaccharides, peptides and proteins, polyphenols, and alkaloids) obtained from microorganisms and marine organisms as a valuable tool to hit various molecular targets against cancer metastasis (Figure 5 and Figure S1).

Considering the ever-growing market for marine biology, the plethora of naturally occurring migrastatic compounds has immense potential to provide numerous clinically relevant anti-migratory and anti-invasive compounds for cancer therapy in the near future. With this comprehensive 2022 update, we recommend the recent knowledge on marine migrastatic compounds to redirect pharmaceutical and scientific research efforts on antimetastatic drugs as endpoints of anti-cancer therapies.

## Figures and Tables

**Table 1:** Marine polysaccharide as migrastatics.

Polysaccharides	Marine Source	Composition	In Vivo Animal Model	Target
Exopolysaccharide 11	*Bacillus* sp. 11	Mannose, glucosamine, galacturonic acid, glucose and xylose, in a ratio of 1:2.58:0.68:0.13:3.09:1.41	Melanoma (mice model)	Filiform structures; cytoskeleton; CD99 [41]
**Polysaccharides**	**Marine source**	**Composition**	**In vitro cell model**	**Target**
Exopolysaccharide 11	*Bacillus* sp. 11	Mannose, glucosamine, galacturonic acid, glucose and xylose, in a ratio of 1:2.58:0.68:0.13:3.09:1.41	Human lung cancer (A549 cells); human hepatoma (Huh7.5, HepG2, Bel-7402 cells)	Filiform structures; cytoskeleton; CD99 [40,41,42]
Fucoidan-like STPC2	*S. thunbergii*	Fucose, xylose, galactose and glucuronic acid, in a ratio of 8.1:3.8:2.1:1.0	Human lung cancer (A549 cells)	MMP-2 [43]
Laminaran sulfate	*F. evanescens*	β-(1→3)-linked D-glucopyranose and single β-(1→6)-linked D-glucose residues. Branches at C6 can be glucose or gentiobiose.	Human colorectal adenocarcinoma (HCT116 cells); human malignant melanoma (SK-MEL-5 cells); human breast adenocarcinoma (MDA-MB-231 cells)	MMP-2/9 [44]
Laminaran sulfate	*D. dichotoma*	(1→3)-linked glucopyranose residues with the branches of single glucose residues at C6 (ratio of bonds 1.3:1.6 = 3:1)	Human melanoma (SK-MEL-28 cells)	MMP-2/9 [45]
SPUP	*U. Pinnatifida*	Fucose, glucose, and galactose, in a ratio of 27.22:19.32:53.46	Human breast cancer (MCF-7 cells); human ovarian cancer (SKOV-3 and A2780 cells)	Apoptosis [46]; Hedgehog signaling pathway [47]
BFP-3	*B. fuscopurpurea*	Rhamnose, arabinose, mannose, glucose, and galactose	Human ovarian cancer (A2780 cells)	Apoptosis, autophagy [49]
Fucoidan	*C. frondosa*	L-fucose and a sulfate ester group. Total carbohydrate content 72.5%; sulfate content 26.1%. (98% purity).	Human osteosarcoma (U2OS cells)	Cytoskeleton; FAK [50]
SIP-SII	*S. maindroni*	Fucose, N-acetylgalactosamine and mannose, in a molar ratio of 2:2:1, with a single branch of glucuronic acid at the C-3 position of mannose.	Human epidermoid carcinoma (KB cells); human ovarian carcinoma (SKOV-3 cells)	EGF-mediated signaling pathway; MMP-2 [51,52]
Sulfated galactans	*G. fisheri*	3-linked β-d-galactopyranose and 4-linked 3,6-anhydro-α-l-galactopyranose or α-l-galactose-6-sulfate with partial methylation at 2-O-methylated-3,6-andydro-α-l-galactopyranose, 6-O-methylated-β-d-galactopyranose and 4-O-methyl-β-l-galactopyranose attached to C-6 of 3-linked-β-d-galactopyranose units, together with sulfation on C-4 and C-6 of d-galactopyranose units.	Human intrahepatic cholangiocarcinoma (HuCCA-1 cells)	EGFR-ERK signaling pathway; FAK; E-cadherin [54,55]
CCB-F0.5	*C. cupressoides* var. *flabellata*	Galactose and mannose in a molar ratio of 1.0:0.1 and traces of xylose. Low protein content 0.17%	Murine melanoma (B16-F10 cells)	*Not described* [56]
CCB-F1.0	*C. cupressoides* var. *flabellata*	Galactose, mannose, and xylose in a molar ratio of 1.0:0.1:0.6 and traces of glucose and rhamnose. Low protein content 0.19%	Murine melanoma (B16-F10 cells)	*Not described* [56]
Crude sulfate polysaccharide extract	*P. tetrastromatica*	-	Human cervical adenocarcinoma (HeLa cells)	MMP-2/9; angiogenesis [57]

**Table 2:** Marine depsipeptides as migrastatics.

Depsipeptides	Marine Source	In Vitro Cell Model	Target
Nobilamide I	*Saccharomonospora* sp., strain CNQ-490	Human lung cancer (A549 cells); human gastric cancer (AGS cells); human colorectal cancer (Caco2 cells)	MMP-2/9 [62]
Molassamide	Cyanobacterium DRTO-73, *Leptolyngbya* sp.	Human breast adenocarcinoma (MDA-MB-231 cells)	ICAM-1 [63]
Chromopeptide A	*Chromobacterium* sp., strain HS-13-94	Human prostate cancer (PC3 cells); mouse PC3 xenograft model	HDACs [66]
Actinomycin V	*Streptomyces* sp., strain N1510.2	Human breast adenocarcinoma (MDA-MB-231 cells)	EMT [68]
CLP	Marine microorganisms	Human breast adenocarcinoma (MDA-MB-231 and MDA-MB-468 cells)	CD151/EGFR signaling [69]
Kempopeptin C	*Lyngbya* sp.	Human breast adenocarcinoma (MDA-MB-231)	*Not described* [70]
Grassystatin F	Cyanobacterium VPG 14-61	Human breast adenocarcinoma (MDA-MB-231)	Protease inhibitor [71]

**Table 3:** The amino acid sequence and molecular mass of marine-derived migrastatic peptides and proteins.

Peptides and Proteins	Marine Source	Sequence	Molecular Mass	In Vitro Cell Model	Target
BABP	*H. discus hannai*	EMDEAQDPSEW	1234.41 Da	Human fibrosarcoma (HT1080 cells); human umbilical vein endothelial (HUVEC cells)	MMPs; VEGF [73]
AATP	*H. discus hannai*	KVEPQDPSEW	1214.30 Da	Fibrosarcoma (HT1080 cells); human umbilical vein endothelial (HUVEC cells)	MMPs; VEGF [74]
αO-conotoxin GeXIVA	*C. generalis*	TCRSSGRYCRSPYDRRRRYCRRITDACV ^1^	3452 Da	Human breast cancer (MDA-MB-157 cells)	EMT [76]
N-V protease (or NAP)	*N. virens*	QAPNYSTASYNVVAVKINLFLSTNNKLYIHDTGVRAVYLAGMKVYLAANPTASSQTFNSDTLVYILDTGINEPNYYINLY ^2^	8888 Da	Human lung cancer (H1299 cells)	apoptosis [78]
PBN11-8	*B. pumilus*	ASTGSQKVTVYAVAD ^3^	19,000 Da	Human hepatocellular carcinoma (BEL-7402 cells)	FAK; MMP-2/9 [79]

^1^ C-terminal COOH sequence of αO-conotoxin GeXIVA. ^2^ N-V protease (No. P83433) in the Swiss-Prot protein sequence database. ^3^ N-terminal partial sequence of PBN11-8. The entire amino acid sequence of PBN11-8 shares 98.5% similarity with peptidase M84 of *Bacillus pumilus* (GenBank accession WP_025208148).

**Table 4:** Marine phenolic compounds as migrastatics.

Phenolic Compound	Marine Source	Chemical Structure	In Vitro Cell Model	Target
7-phloroeckol	*E. cava*	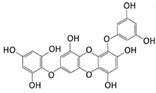	Human hepatoma cancer (HepG2 cells); human umbilical vein endothelial (HUVEC cells)	HIF-1α; angiogenesis [84]
Dieckol	*E. cava*	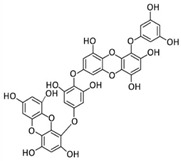	Human breast cancer (MCF-7, MDA-MB-231 cells)	MMP-2/9; TLR-4; NF-κB [85]
Phlorofucofuroeckol	*E. cava*	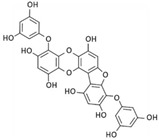	Human breast cancer (MCF-7, MDA-MB-231 cells)	MMP-2/9; TLR-4; NF-κB [85]
Dihydroauroglaucin	*E. chevalieri*	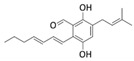	Human neuroblastoma (SH-SY5Y cells)	Autophagy [87]
Viriditoxin	*P. variotii*	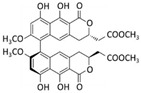	Human ovarian cancer (SKOV-3 cells)	Cytoskeleton [89]
Gallic acid	*P. boergeseni*, *P. oceanica*	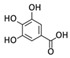	Human renal cancer (A498, ACHN cells); human fibrosarcoma (HT1080 cells); human neuroblastoma (SH-SY5Y cells)	Apoptosis [91]; autophagy [93]; autophagy, MMP-2 [94]
Caffeic acid	*P. boergeseni*	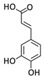	Human renal cancer (A498, ACHN cells)	Apoptosis [91]
Rutin	*P. boergeseni*	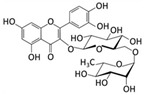	Human renal cancer (A498, ACHN cells)	Apoptosis [91]
Quercetin	*P. boergeseni*	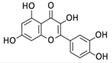	Human renal cancer (A498, ACHN cells)	Apoptosis [91]
Ferulic acid	*P. boergeseni*, *P. oceanica*	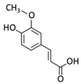	Human renal cancer (A498, ACHN cells); human fibrosarcoma (HT1080 cells); human neuroblastoma (SH-SY5Y cells)	Apoptosis [91]; autophagy [93]; autophagy, MMP-2 [94]
Catechin	*P. oceanica*	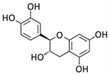	Human fibrosarcoma (HT1080 cells); human neuroblastoma (SH-SY5Y cells)	Autophagy [93]; autophagy, MMP-2 [94]
Epicatechin	*P. oceanica*	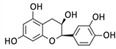	Human fibrosarcoma (HT1080 cells); human neuroblastoma (SH-SY5Y cells)	Autophagy [93]; autophagy, MMP-2 [94]
Chlorogenic acid	*P. oceanica*	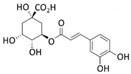	Human fibrosarcoma (HT1080 cells); human neuroblastoma (SH-SY5Y cells)	Autophagy [93]; autophagy, MMP-2 [94]

**Table 5:** Marine alkaloids as migrastatics.

Alkaloids	Marine Source	In Vitro Cell Model	Target
Aeroplysinin-1	*A. aerophoba*	Mouse pheocromocytoma cells (MTT cells)	Integrin β1 [97]
Manzamine A	*Haliclona* sp., *Xestospongia* sp. and *Pellina* sp.	Human colorectal carcinoma (HCT116 cells)	EMT [98]
Jorunnamycin A	*Xestospongia* sp.	Human lung cancer (H460 cells)	EMT; anoikis [100]
Normonanchocidines G and H	*M. pulchra*	Human cervical carcinoma (HeLa cells)	*Not described* [101]

## Supplementary Materials

The following supporting information can be downloaded at: https://www.mdpi.com/article/10.3390/md20050273/s1. Figure S1: Schematic representation of potential molecular targets of marine migrastatics (2017–2022).
