# Supplementary material for: Marine Migrastatics: A Comprehensive 2022 Update
Source: Mar Drugs. 2022 Apr 19;20(5):273. doi: 10.3390/md20050273 (PMC9145002; doi:10.3390/md20050273)
Supplement: Supplementary file 1 [file marinedrugs-20-00273-s001.zip › marinedrugs-1672861-supplementary.pdf]

Click over pictures

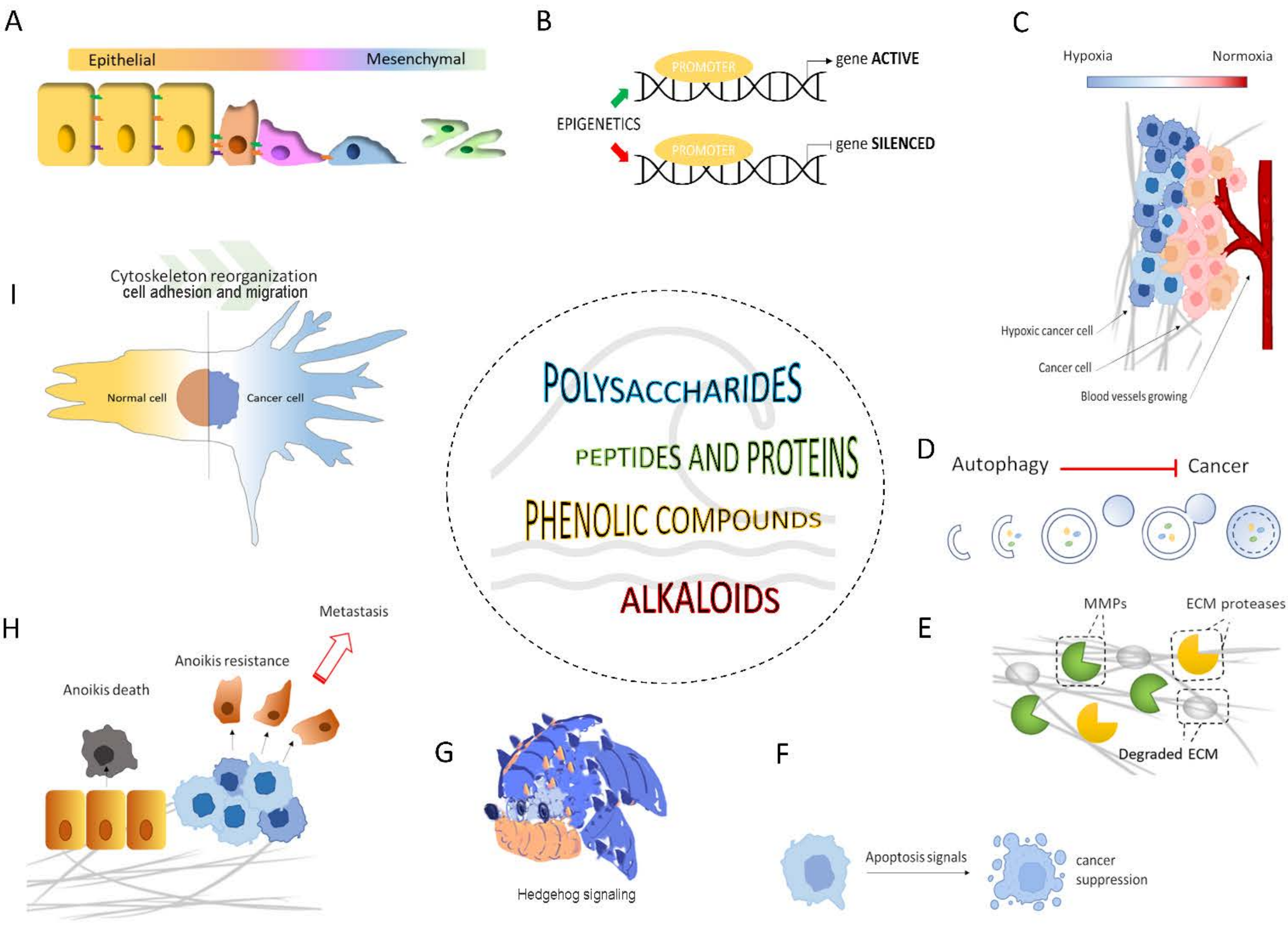

Figure S1. Schematic representation of potential molecular targets of marine migrastatics (2017-2022): (A) epithelial-mesenchymal transition; (B) epigenetic control; (C) neo-angiogenesis and hypoxia; (D) autophagy; (E) ECM degradation by MMPs and other proteases; (F) apoptosis; (G) Hedgehog signalling pathway; (H) resistance to anoikis; (I) cytoskeletal reorganization, cell adhesion and migration. Click on each picture to view the involved migrastatic marine compound.

To go back to the pictures click on the table

Epithelial-to-Mesenchymal Transition

Compound	Marine source
**Actinomycin V**	***Streptomyces sp.***
**Jorunnamycin A**	***Xestospongia sp.***
**Manzamine A**	***Haliclona sp., Xestospongia sp. Pellina sp.***
**αO-conotoxin GeXIVA**	***C. generalis***

To go back to the pictures click on the table

Epigenetic control

Compound	Marine source
**Chromopeptide A**	***Chromobacterium sp., HS-13-94***

To go back to the pictures click on the table

Neo-angiogenesis and hypoxia

Compound	Marine source
**AATP**	***H. discus hannai***
**BABP**	***H. discus hannai***
**Crude sulfate polysaccharides extract**	***P. tetrastromatica***
**7-Phloroeckol**	***E. Cava***

To go back to the pictures click on the table

Autophagy

Compound	Marine source
**BFP-3**	***B. fuscopurpurea***
**Catechin**	***P. Oceanica***
**Chlorogenic acid**	***P. Oceanica***
**Dihydroauroglaucin**	***E. chevalieri***
**Epicatechin**	***P. Oceanica***
**Ferulic acid**	***P. boergeseni, P. oceanica***
**Gallic acid**	***P.. boergeseni, P. oceanica***

To go back to the pictures click on the table

ECM degradation by MMPs and other proteases

Compound	Marine source
AATP	*H. discus hannai*
BABP	*H. discus hannai*
Catechin	*P. oceanica*
Chlorogenic acid	*P. Oceanica*
Crude sulfate polysaccharides extract	*P. tetrastromatica*
Dieckol	*E. Cava*
Epicatechin	*P. Oceanica*
Ferulic acid	*P. boergeseni, P. oceanica,*
Fucoidan-like STPC2	*S. thunbergii*
Gallic acid	*P. boergeseni, P. oceanica*
Grassystatin F	*Cyanobacterium VPG 14-61*
Laminaran sulfate	*F. evanescens*
Nobilamide I	*Saccharomonospora sp., strain CNQ-490*
PBN11-8	*B. pumilus*
Phlorofucofuroeckol	*E. cava*
SIP-SII	*S. maindroni*

To go back to the pictures click on the table

Apoptosis

Compound	Marine source
BFP-3	*B. fuscopurpurea*
Caffeic acid	*P. boergeseni*
Ferulic acid	*P. boergeseni, P. oceanica*
Gallic acid	*P. boergeseni, P. oceanica*
N-V protease (or NAP)	*N. virens*
Quercetin	*P. boergeseni*
Rutin	*P. boergeseni*

To go back to the pictures click on the table

Hedgehog signaling

Compound	Marine source
**SPUP**	***U. Pinnatifida***

To go back to the pictures click on the table

Resistance to anoikis

Compound	Marine source
**Jorunnamycin A**	***Xestospongia sp.***

To go back to the pictures click on the table

Cytoskeletal reorganization, cell adhesion and migration

Compound	Marine source
**Aeroplysinin-1**	***A. aerophoba***
**Chromopeptide A**	***Chromobacterium sp., HS-13-94***
**CLP**	***Marine microorganisms***
**Dieckol**	***E. Cava***
**Exopolysaccharide 11**	***Bacillus sp. 11***
**Fucoidan**	***C. frondosa***
**Molassamide**	***Cyanobacterium DRTO-73***
**PBN11-8**	***B. pumilus***
**Phlorofucofuroeckol**	***E. Cava***
**SIP-SII**	***S. maindroni***
**Sulfated galactans**	***G. fisheri***
**Viriditoxin**	***P. variotii***
